# Secreted Ectodomain of SIGLEC-9 and MCP-1 Synergistically Improve Acute Liver Failure in Rats by Altering Macrophage Polarity

**DOI:** 10.1038/srep44043

**Published:** 2017-03-08

**Authors:** Takanori Ito, Masatoshi Ishigami, Yoshihiro Matsushita, Marina Hirata, Kohki Matsubara, Tetsuya Ishikawa, Hideharu Hibi, Minoru Ueda, Yoshiki Hirooka, Hidemi Goto, Akihito Yamamoto

**Affiliations:** 1 Department of Gastroenterology and Hepatology, 65 Tsurumai-cho, Showa-ku, Nagoya 466-8550, Japan; 2 Department of Oral and Maxillofacial Surgery of Nagoya University Graduate School of Medicine, 65 Tsurumai-cho, Showa-ku, Nagoya 466-8550, Japan; 3 Department of Oral histology, Institute of Biomedical Science, Tokushima University Graduate School, 3-18-5 Kuramoto-cho, Tokushima 770-8504, Japan

## Abstract

Effective treatments for acute liver failure (ALF) are still lacking. We recently reported that a single intravenous administration of serum-free conditioned medium from stem cells derived from human exfoliated deciduous teeth (SHED-CM) into the D-galactosamine (D-Gal)-induced rat ALF model improves the liver injury. However, the specific factors in SHED-CM that are responsible for resolving ALF remain unclear. Here we found that depleting SHED-CM of two anti-inflammatory M2 macrophage inducers—monocyte chemoattractant protein-1 (MCP-1) and the secreted ectodomain of sialic acid-binding Ig-like lectin-9 (sSiglec-9)—abolished its ability to resolve rat ALF. Furthermore, treatment with MCP-1/sSiglec-9 alone dramatically improved the survival of ALF rats. This treatment induced anti-inflammatory M2, suppressed hepatocyte apoptosis, and promoted hepatocyte proliferation. Treatment with an M2-depletion reagent (mannosylated clodronate liposomes) suppressed the recovery. In addition, MCP-1 and sSiglec-9 synergistically promoted the M2 differentiation of bone marrow-derived macrophages via CCR2, accompanied by the production of multiple liver-regenerating factors. The conditioned medium from MCP-1/sSiglec-9-activated M2 macrophages, but not from interleukin-4-induced ones, suppressed the D-Gal- and LPS-induced apoptosis of primary hepatocytes and promoted their proliferation *in vitro*. The unique combination of MCP-1/sSiglec-9 ameliorates rat ALF by inhibiting hepatocellular apoptosis and promoting liver regeneration through the induction of anti-inflammatory/tissue-repairing M2 macrophages.

In acute liver failure (ALF), a poorly controlled inflammatory response causes extensive hepatic destruction, which leads to systemic inflammation, multiple organ failure, and sudden death^1^,^2^. Although there are some supportive treatments, such as blood purification, recovery from ALF depends on inherent hepatic regenerative activities. However, there are no effective interventions to promote hepatic regeneration. Liver transplantation is currently the only available treatment, but its application is limited due to the shortage of donors and exorbitant cost. Therefore, alternative treatments for patients with ALF are urgently needed^3^.

Hepatic macrophages play critical roles in the pathophysiology of ALF^4^,^5^,^6^. Macrophages are a heterogeneous population of immune cells consisting of several subtypes, including the pro-inflammatory M1- and anti-inflammatory M2-type macrophages^7^,^8^,^9^. Classically activated M1 cells initiate inflammation and accelerate tissue destruction by releasing high levels of pro-inflammatory cytokines, reactive oxygen species, and nitric oxide, whereas M2 cells counteract pro-inflammatory M1 conditions by secreting anti-inflammatory cytokines, scavenging cellular debris, and suppressing fibrosis. The balance of these polarized macrophages is thought to be important for tissue repair and regeneration^7^,^10^. In ALF, however, large numbers of M1 cells that rarely convert to M2 cells are continuously produced, leading to irreversible tissue destruction in the liver^11^. Thus, strategies designed to control M1/M2 polarity in the early stages of ALF may provide significant therapeutic benefits.

Stem cells derived from human exfoliated deciduous teeth (SHEDs) are a population of self-renewing mesenchymal stromal cells (MSCs)^12^. We recently reported that a single intravenous administration of SHEDs or SHED-derived serum-free conditioned medium (SHED-CM) into the D-galactosamine (D-Gal)-induced rat model of ALF, markedly improves the liver injury and survival rates. SHED-CM attenuates the ALF-induced pro-inflammatory response and generates an anti-inflammatory environment, accompanied by the induction of anti-inflammatory M2-like hepatic macrophages^13^. We analyzed the secretome of SHED-CM and identified a novel set of anti-inflammatory M2 macrophage inducers: monocyte chemoattractant protein-1/CC chemokine ligand 2 (MCP-1/CCL2) and the secreted ectodomain of sialic acid-binding Ig-like lectin-9 (sSiglec-9)^14^. MCP-1 is a chemokine that recruits immune cells to inflamed tissues^15^, and Siglecs are a large family of sialic-acid-binding type-I transmembrane immunoglobulin-like lectins that modulate the immune signaling on various types of immune cells^16^. Notably, the intrathecal administration of MCP-1 and sSiglec-9 synergistically repairs the injured rat spinal cord through the induction of anti-inflammatory M2-like macrophages and improves neurological deficits^14^. However, the therapeutic potential of MCP-1/sSiglec-9 for ALF has not been evaluated. In this study, we investigated the roles of MCP-1/sSiglec-9 in the SHED-CM-mediated recovery from rodent ALF and their therapeutic potential for ALF.

## Results

### SHED-CM lacking both MCP-1 and sSiglec-9 fails to induce M2 macrophages or to promote recovery from ALF

The SHEDs used in this study exhibited a fibroblastic morphology with a bipolar spindle shape, expressed MSC markers (CD90, CD73, and CD105), but not endothelial/hematopoietic markers (CD34, CD45, CD11b/c, or HLA-DR), and were capable of undergoing adipogenic, chondrogenic, and osteogenic differentiation^17^. There was no significant difference in the cellular survival of the SHEDs after their incubation in serum-free versus serum-containing DMEM (data not shown).

**Figure 1:**
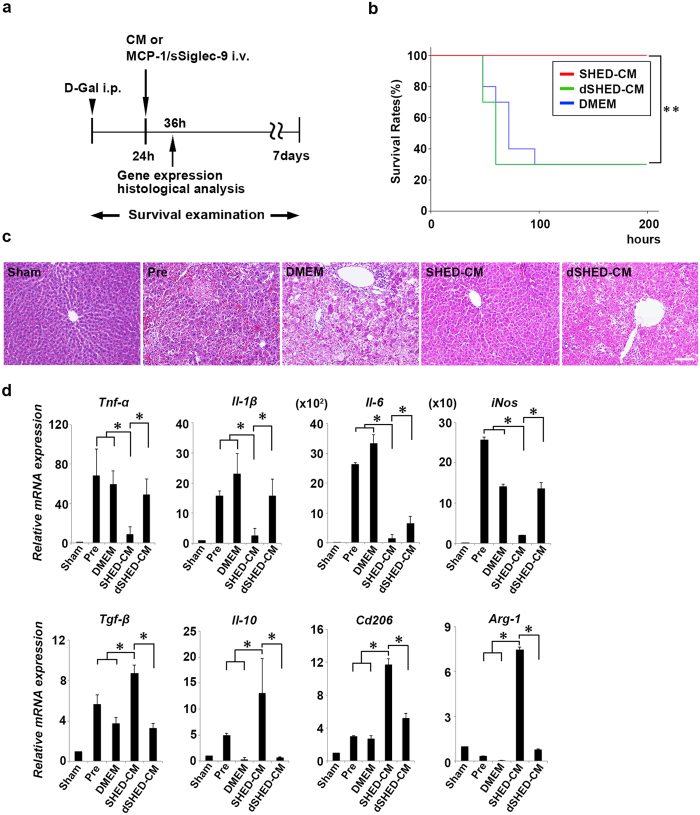
MCP-1 and sSiglec-9 are essential for SHED-CM-mediated M2 induction and recovery from ALF. (**a**) Experimental design. D-Gal, D-galactosamine; CM, conditioned medium; MCP-1, monocyte chemoattractant protein-1; sSiglec-9, secreted ectodomain of sialic acid-binding Ig-like lectin-9; i.v., intravenous injection; i.p., intraperitoneal injection. (**b**) Kaplan–Meier survival analysis of SHED-CM, dSHED-CM, and DMEM treatment groups. The log rank test was used to compare the SHED-CM (n = 11) and dSHED-CM (n = 10) groups (*P* = 0.002), and the SHED-CM and DMEM (n = 10) groups (*P* = 0.002). ∗∗*P* < 0.01. (**c**) Representative images of HE-stained livers. Scale bar: 100 μm. Pre, pre-treatment. (**d**) Levels of the indicated mRNAs in the livers of CM-treated rats. Results are expressed relative to the level in the sham-operated model. Data represent the mean ± standard error of the mean (SEM) (n = 4 per group); ∗*P* < 0.05.

MCP-1 and sSiglec-9 were specifically immunodepleted from the SHED-CM (dSHED-CM), and their loss was confirmed by Enzyme-Linked ImmunoSorbent Assay (ELISA) (Supplementary Table S1). Rat ALF was induced by the intraperitoneal injection of D-Gal. Four days later, 60% of the rats had died, and the surviving ones exhibited severe liver damage with a large infiltration of mononuclear cells. In contrast, rats receiving a single intravenous administration of SHED-CM 24 h after D-Gal injection exhibited markedly reduced liver damage and increased survival rates. Notably, rats receiving dSHED-CM did not show improved liver damage or survival rates (Fig. 1b,c).

Quantitative RT-PCR analysis revealed that the SHED-CM treatment strongly suppressed the mRNA expression of pro-inflammatory mediators (*Tnf-α, Il-1β, Il-6*, and *iNos*) and increased the expression of anti-inflammatory M2 markers (*Tgf-β, Il-10, Cd206*, and *Arg-1*) (Fig. 1d). In contrast, dSHED-CM treatment failed to reduce the expression of pro-inflammatory M1 markers or to induce the expression of anti-inflammatory M2 markers. Taken together, these results suggested that MCP-1 and sSiglec-9 in the SHED-CM are essential for M2 macrophage activation and the resolution of D-Gal-induced liver injury.

### MCP-1 and sSiglec-9 synergistically induce M2 macrophages via CCR2, accompanied by the production of multiple trophic factors

**Figure 2:**
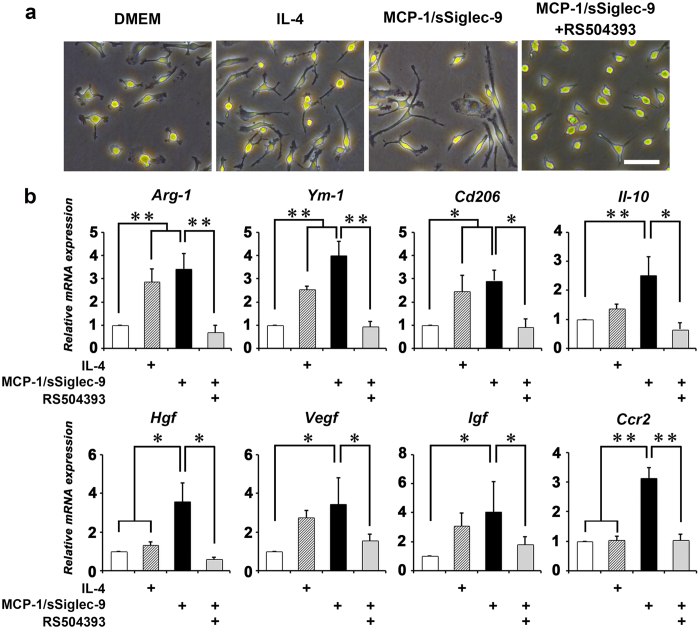
MCP-1 and sSiglec-9 activate M2-like macrophage differentiation *in vitro*. (**a**) Phase contrast images of bone marrow macrophages (BMMs) cultured with DMEM, IL-4, MCP-1/sSiglec-9, alone or together with the CCR2 inhibitor, RS504393. Scale bar: 100 μm. (**b**) Relative mRNA expression levels in the treated BMMs (n = 4 per group). Data represent the mean ± SEM; ∗*P* < 0.05, ∗∗*P* < 0.01.

Bone marrow macrophages (BMMs) treated with MCP-1/sSiglec-9 or IL-4 exhibited the hallmark characteristics of M2 macrophages: an elongated cell shape and increased expression of M2 markers (*Arg-1, Ym-1, Cd206,* and *Il-10*) (Fig. 2a,b)^18^. Furthermore, the MCP-1/sSiglec-9-treated BMMs showed significantly increased gene expression of multiple trophic factors for liver regeneration (*Hgf, Vegf,* and *Igf*) compared with control BMMs, and significantly higher *Hgf* expression and a tendency toward higher levels of *Il-10, Vegf,* and *Igf* expression than the IL-4-treated BMMs. Treating BMMs with the selective CCR2 antagonist RS504393 inhibited the MCP-1/sSiglec-9-induced expression of both the M2 markers and the trophic factors (Fig. 2b).

### MCP-1/sSiglec-9-induced M2 macrophages protect cultured rat hepatocytes from apoptosis and induce their proliferation

**Figure 3:**
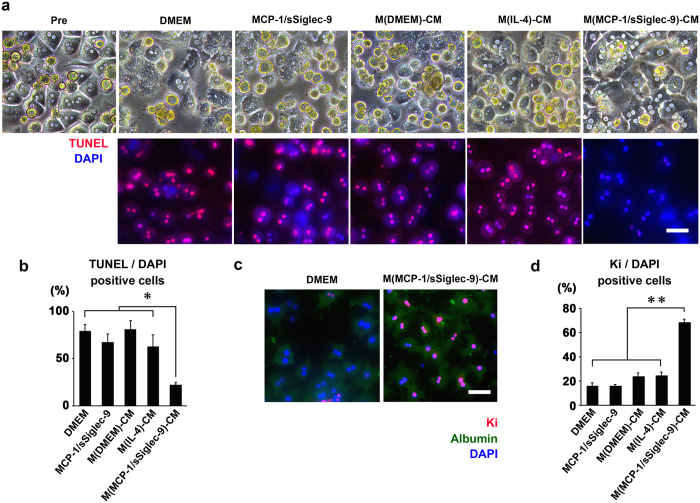
CM from MCP-1/sSiglec-9-induced M2 macrophages protects hepatocytes from apoptosis and induces their proliferation under D-gal and LPS stimulation *in vitro*. (**a**) Phase contrast images of hepatocytes stimulated with D-Gal and lipopolysaccharide (LPS) for 24 h in DMEM containing MCP-1 and sSiglec-9 proteins, M(DMEM)-CM, M(IL-4)-CM, or M(MCP-1/sSiglec-9)-CM (upper). Representative images of TUNEL-stained hepatocytes that were stimulated as above (lower). Scale bar: 100 μm. (**b**) Quantification of TUNEL^+^/DAPI-stained hepatocytes. Data represent the mean ± SEM; ∗*P* < 0.05. (**c**) Representative images of Ki- and Albumin-stained hepatocytes stimulated with D-Gal and LPS for 24 h in M(MCP-1/sSiglec-9)-CM. Scale bar: 100 μm. (**d**) Quantification of the Ki^+^/DAPI-stained hepatocytes. Data represent the mean ± SEM; ∗∗*P* < 0.01.

Next, we examined the hepatocyte regenerative activity of MCP-1/sSiglec-9- or IL-4-induced BMMs by evaluating the activities of their conditioned medium (CM) *in vitro*. Primary hepatocytes stimulated with D-Gal and LPS underwent apoptosis. While the addition of MCP-1/sSiglec-9 protein, M(DMEM)-CM, or M(IL-4)-CM to the hepatocytes had little or no effect on the D-Gal/LPS-induced apoptosis, treating hepatocytes with M(MCP-1/sSiglec-9)-CM significantly suppressed it (Fig. 3a,b). Furthermore, the number of Ki-67^+^/Albumin^+^ proliferating hepatocytes in the M(MCP-1/sSiglec-9)-CM group was significantly higher than that in the other groups (Fig. 3c,d). The mean numbers of TUNEL^+^ and Ki-67^+^/DAPI^+^ hepatocytes are shown in Supplementary Table S2.

### A single intravenous administration of MCP-1/sSiglec-9 into the rat promotes recovery from ALF

**Figure 4:**
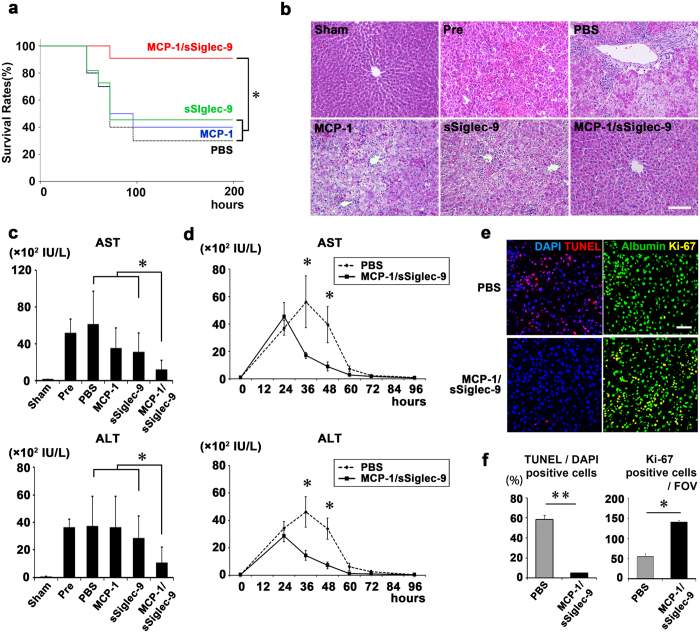
Therapeutic benefits of MCP-1 and sSiglec-9 for ALF in rats. (**a**) Kaplan–Meier survival analysis of the MCP-1 and/or sSiglec-9, and PBS-treated groups. The log rank test was used to compare the MCP-1/sSiglec-9 and MCP-1 groups (*P* = 0.04), the MCP-1/sSiglec-9 and sSiglec-9 groups (*P* = 0.02), and the MCP-1/sSiglec-9 and PBS groups (*P* = 0.007). ∗*P* < 0.05, n = 10 per group. (**b**) Representative images of HE-stained livers; Scale bar: 100 μm. (**c**) Quantification of serum liver enzymes 24 h after MCP-1 and/or sSiglec-9 administration. Data represent the mean ± SEM; ∗*P* < 0.05. (**d**) Serum transaminase levels over time after D-Gal injection in MCP-1/sSiglec-9 and PBS treatment groups. Data represent the mean ± SEM; ∗*P* < 0.05. (**e**,**f**) Representative images of TUNEL and Albumin/Ki-67 immunofluorescence staining of the livers, 12 h after MCP-1/sSiglec-9 injection. Quantification of the ratio between TUNEL-positive and DAPI-stained nuclei (left, data represent the mean ± SEM; ∗∗*P* < 0.01), and the number of Ki-67-positive hepatocytes (right, data represent the mean ± SEM; ∗*P* < 0.05) by digital image analysis. FOV, field of view. Scale bar: 50 μm.

Next, we examined the therapeutic effects of MCP-1/sSiglec-9 for rat ALF. Twenty-four hours after D-Gal injection (Pre), we observed increased peripheral blood liver enzyme (AST and ALT) levels that peaked 36 h after D-Gal injection. Notably, a single intravenous administration of MCP-1/sSiglec-9, but not of MCP-1 or sSiglec-9 alone, at 24 h after D-Gal injection markedly decreased the AST and ALT levels (Fig. 4c,d) and improved survival rates compared with the PBS-treated group (Fig. 4a, *p < *0.01).

Histological examination revealed a large infiltration of mononuclear cells, along with severe hepatic lobule disorganization, hepatic cord disorders, and extensive hepatic destruction in the MCP-1-alone, sSiglec-9-alone, and PBS-treated groups. In contrast, the MCP-1/sSiglec-9-treated group exhibited only mild inflammation and a relatively normal hepatic morphology (Fig. 4b). MCP-1/sSiglec-9-treated rats also had significantly lower numbers of TUNEL^+^ apoptotic hepatocytes than the PBS-treated rats. The numbers of Ki-67^+^/Albumin^+^ proliferating hepatocytes in the MCP-1/sSiglec-9-treated group was approximately 3 times that in the PBS-treated group (Fig. 4e,f). The numbers of TUNEL^+^/DAPI^+^ and Ki-67^+^ cells are shown in Supplementary Table S3. These results demonstrated that MCP-1/sSiglec-9 treatment restored liver function in ALF rats and activated regenerative mechanisms.

### MCP-1/sSiglec-9 treatment suppresses the ALF-induced pro-inflammatory M1 response and promotes an anti-inflammatory M2 response

**Figure 5:**
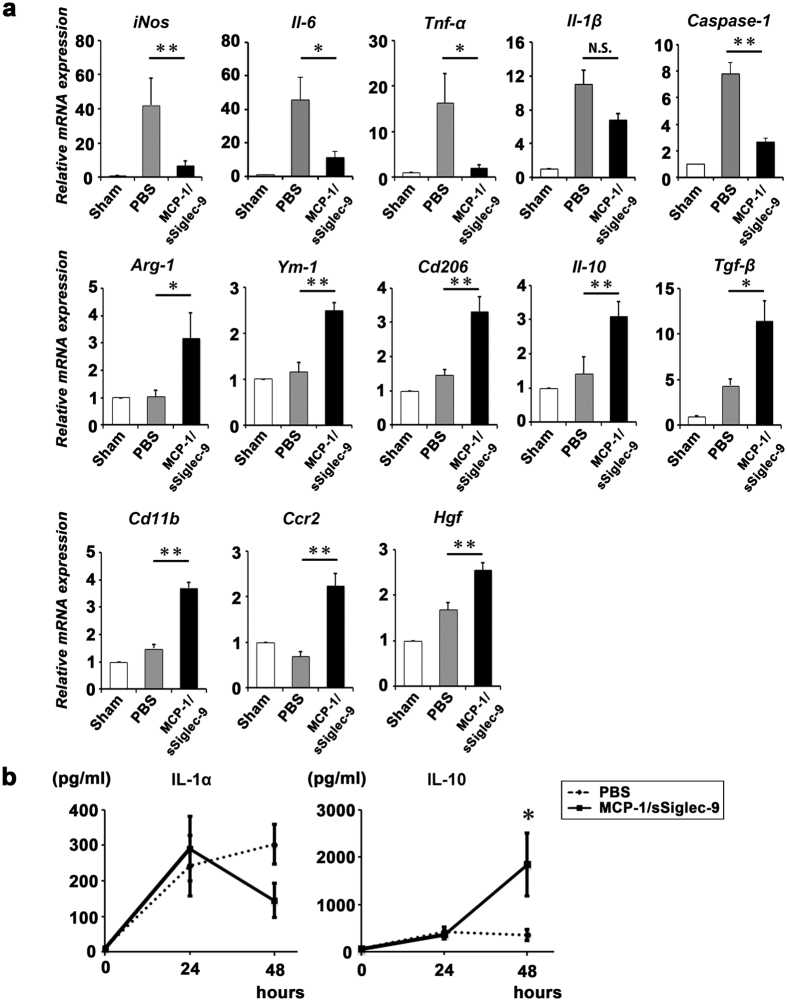
MCP-1/sSiglec-9 suppresses inflammation and induces an anti-inflammatory M2 response in ALF in rats. (**a**) Gene expression in the livers of PBS- and MCP-1/sSiglec-9-treated rats 12 h after MCP-1/sSiglec-9 injection (n ≥ 4 animals per group). The mRNA results are expressed relative to the level in the sham-operated model. Data represent the mean ± SEM; ∗*P* < 0.05, ∗∗*P* < 0.01. N.S., not significant. (**b**) Levels of circulating inflammatory cytokines in the PBS- and MCP-1/sSiglec-9 treatment groups. Serum samples were collected 24 (Pre) and 48 h after D-Gal injection (Sham n = 2, PBS n = 5, MCP-1/sSiglec-9 n = 4). Data represent the mean ± SEM; ∗*P* < 0.05.

Quantitative RT-PCR analysis revealed that MCP-1/sSiglec-9 treatment strongly suppressed the expressions of pro-inflammatory and apoptosis mediators, but increased M2 markers (Fig. 5a). In addition, *Hgf*, which encodes a prominent factor involved in hepatocyte protection and regeneration, was significantly up-regulated in the MCP-1/sSiglec-9-treated group. We also found that the serum concentration of the anti-inflammatory cytokine IL-10 was approximately 6 times higher after MCP-1/sSiglec-9 treatment than before (p = 0.04), and that the MCP-1/sSiglec-9-treated group tended to express lower levels of the pro-inflammatory cytokine, IL-1α than the PBS-treated group (Fig. 5b).

**Figure 6:**
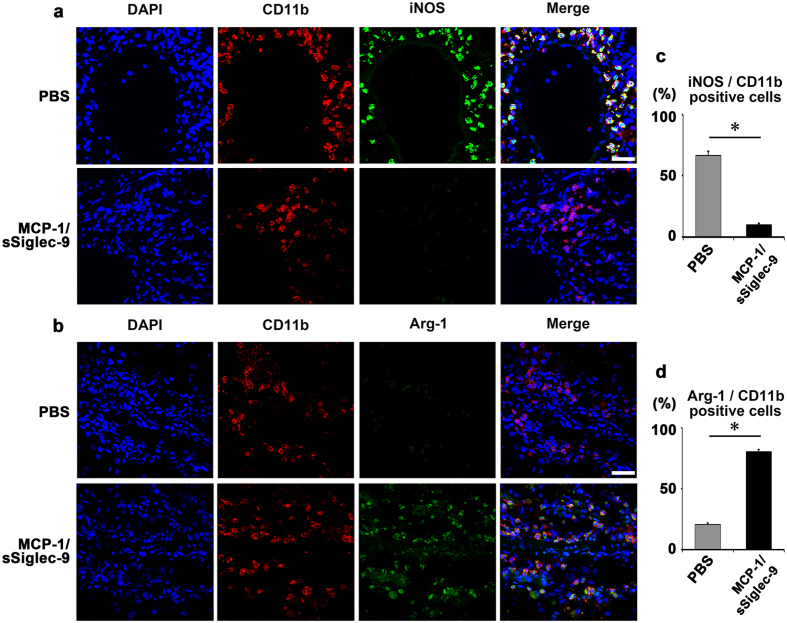
MCP-1/sSiglec-9 promotes the M2 differentiation of hepatic macrophages. Representative images of immunohistologically stained rat liver macrophages 12 h after MCP-1/sSiglec-9 injection. Note that the CD11b^+^ hepatic macrophages expressed both iNOS (**a**) and Arginase-1 (**b**) Scale bar: 200 μm. Quantification of the total number of iNOS^+^/CD11b^+^ cells (**c**) and Arginase-1^+^/CD11b^+^ cells (**d**) Samples from three animals per treatment group were analyzed. Data represent the mean ± SD; ∗*P* < 0.05. Scale bar: 200 μm.

Consistent with these observations, immunohistochemical staining revealed that the percentage of Arginase-1^+^ CD11b^+^ M2 cells was increased, while that of iNOS^+^CD11b^+^ M1 cells was decreased in the MCP-1/sSiglec-9-treated liver compared to the PBS-treated liver (Fig. 6). The numbers of iNOS^+^CD11b^+^ and Arginase-1^+^CD11b^+^ cells are shown in Supplementary Table S4.

Liver regeneration is also reported to be dependent on hepatic stellate cell (HSC) activation^19^,^20^. We therefore investigated whether HSCs played a role in the therapeutic effect in this model. An immunohistological analysis of Desmin, a marker of activated HSCs, showed that the shape and proportion of Desmin-positive HSCs were comparable between the MCP-1/sSiglec-9-treated and PBS-treated livers (Supplementary Fig. S1).

### MCP-1/sSiglec-9-mediated recovery from ALF requires M2 macrophages

**Figure 7:**
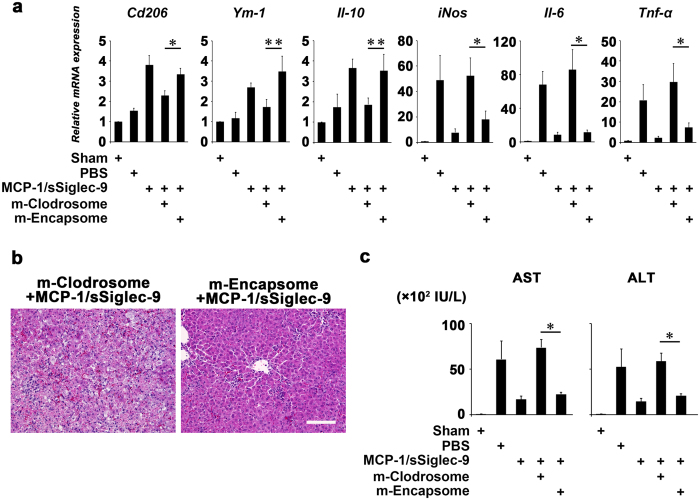
Effects of M2 depletion on the MCP-1/sSiglec-9-mediated recovery from ALF in rats. (**a**) Gene expressions in ALF livers treated with MCP-1/sSiglec-9 together with m-Clodrosome or m-Encapsome 36 h after D-Gal injection (n ≥ 4 animals per group). The results are expressed relative to the mRNA expression level in the sham-operated model. Data represent the mean ± SEM; ∗*P* < 0.05, ∗∗*P* < 0.01. m-Clodrosome, Mannosylated Clodronate liposomes; m-Encapsome, Mannosylated control liposomes. (**b**) Representative images of HE-stained livers. Treatment with m-Clodrosome abolished the MCP-1/sSiglec-9-mediated recovery from ALF. Scale bar: 100 μm. (**c**) Quantification of serum liver enzymes at 36 h after D-Gal injection. Data represent the mean ± SEM; ∗*P* < 0.05.

Finally, we depleted M2 macrophages by m-Clodrosome and examined the effect on the MCP-1/sSiglec-9-mediated recovery from ALF. Treatment with m-Clodrosome, but not with control liposome (m-Encapsome), prevented the MCP-1/sSiglec-9-mediated conversion from the pro-inflammatory M1 condition to the anti-inflammatory M2 one (Fig. 7a). Notably, m-Clodrosome prevented the MCP-1/sSiglec-9-mediated improvement of the hepatic inflammation and damage (Fig. 7b,c).

## Discussion

We previously reported that SHEDs, a type of MSC, secrete a broad repertoire of trophic and immunomodulatory factors, and that a single administration of either SHEDs or SHED-CM protects rats from D-Gal-induced ALF; however, the factors that mediate this protection have been unclear. Here we show that the induction of M2 macrophages by MCP-1/sSiglec-9 in SHED-CM is essential for the SHED-CM-mediated improvement of rat ALF. Notably, we found that a single intravenous administration of the MCP-1/sSiglec-9 markedly attenuated the liver injury and improved the survival rate in ALF rats. Treatment with m-Clodrosome, which specifically eliminates M2 macrophages, abolished the MCP-1/sSiglec-9-mediated recovery from ALF. Furthermore, we showed that the MCP-1/sSiglec-9-induced M2 macrophages expressed multiple trophic factors that promote liver regeneration. M(MCP-1/sSiglec-9)-CM directly suppressed the apoptosis of primary hepatocytes and promoted their proliferation. This is the first report, to our knowledge, demonstrating the remarkable therapeutic benefits of MCP-1/sSiglec-9 for ALF.

The M1/M2 polarization state of the intrahepatic macrophages is thought to reflect the condition of liver injury and recovery^21^. In a recent study, M2 macrophages induced by MSC allo-transplantation were shown to reduce concanavalin A (ConA)-induced liver injury^22^. Furthermore, we previously reported that the transplantation or intravenous injection of SHEDs or SHED-CM accelerates the macrophage M1-to-M2 transition in various animal models of inflammatory disease, including ALF^13^,^14^,^23^,^24^. Collectively, these findings suggest that M1/M2 conversion may be a common mechanism that underlies MSC-mediated tissue repair. However, the clinical use of MSCs is associated with ethical and technical issues; thus, identification of the critical factors involved in MSC-induced M2 polarization might provide a more feasible therapeutic approach. Our finding that MCP-1 and sSiglec-9 could substitute for SHED-CM in treating rat ALF may prove advantageous for treating human ALF, since these factors can be easily produced and rapidly administered to patients.

We also found that M(MCP-1/sSiglec-9)-CM, but not a direct addition of MCP-1/sSiglec-9, significantly suppressed the D-Gal/LPS-induced apoptosis of hepatocytes and induced their proliferation *in vitro*. Furthermore, we found that the MCP-1/sSiglec-9-induced M2 macrophages exhibited increased expressions of mRNAs encoding the hepatoregenerative factors HGF, VEGF, and IGF. HGF is a prominent growth factor involved in hepatocyte proliferation, and functions as a tissue-repairing factor in various rodent disease models, including ALF^25^,^26^,^27^. VEGF is a well-known pro-angiogenic factor that is essential for vascular formation after liver injury^28^. IGF suppresses hepatocyte apoptosis in mouse ALF^29^. Taken together, these data suggest that MCP-1 and sSiglec-9 exert multifaceted tissue-repairing activities by inducing M2-type macrophage polarization.

On the other hand, even though IL-4 is a strong M2 inducer^7^,^9^, M(IL-4)-CM failed to suppress D-Gal/LPS-induced apoptosis in the hepatocyte protection assay. Both the IL-4- and MCP-1/sSiglec-9-induced macrophages expressed similar sets of M2 genes, including *Cd206, Arginase-1,* and *Ym-1;* however, the MCP-1/sSiglec-9-induced macrophages expressed significantly higher *Hgf* levels and tended to express higher levels of *Il-10, Vegf,* and *Igf* than the IL-4-induced macrophages. M1 and M2 macrophages are highly plastic, and neither M1 nor M2 macrophages are homogeneous cell populations^9^. Thus, the MCP-1/sSiglec-9-induced macrophages may represent a distinct population of M2 cells that express higher levels of hepatoregenerative factors than IL-4-induced macrophages. Further studies comparing the transcriptome signatures of the various M2-like populations induced by other known M2 polarizing agents (e.g., IL-4/-13, IL-10, CSF-1) are needed to clarify the unique properties and therapeutic potentials of these cell populations.

Recently, HSCs and Kupffer cells were shown to interact either directly or indirectly through fenestrated endothelium^30^.In addition, Fujita *et al*. reported that HSCs play a key role in controlling the acute hepatic inflammation in ConA–induced hepatitis^31^. Therefore, we investigated whether HSCs influence the MCP-1/sSiglec-9-mediated recovery from ALF *in vivo*. Immunohistological analysis showed that there was little or no difference in HSC activation between the MCP-1/sSiglec-9- and PBS-treated groups. This result indicated that the HSCs’ involvement in the therapeutic effect of MCP-1/sSiglec-9 was limited in this model.

MCP-1 is generally known to recruit monocytes into inflamed tissues^15^,^21^; therefore, therapeutics targeting MCP-1 or CCR2 have been developed to treat several acute and chronic inflammatory diseases^32^. In contrast, LPS-induced endotoxemia in mice was shown to be protected by MCP-1 administration, which increases plasma IL-10 levels, and to be exacerbated by anti-MCP-1 antibody administration, which increases the peak TNF-α and IL-12 levels^33^. With respect to ALF, CCR2^−/−^ mice exhibit increased hepatic injury, with elevated IFN-γ and TNF-α expressions, in an acetaminophen-induced hepatitis model^34^. However, another study reported that CCR2^−/−^ mice exhibit a weakened hepatic inflammatory response to acetaminophen, with a reduced number of infiltrating macrophages and a decreased expression of pro-inflammatory cytokines^35^. These reports indicate that MCP-1/CCR2 may play both detrimental and beneficial roles in the pathophysiology of ALF. Our current study suggests that the second signal, sSiglec-9 in this study, may modulate the pathophysiological roles of MCP-1/CCR2 signaling in the hepatic inflammatory environment.

Although Siglecs are expressed on cells of the innate immune system and regulate immune cell activity via interactions with sialic acids^36^, the functional relationship between Siglecs and the hepatic environment has not been reported. Mouse and rat Siglec-E, which have three extracellular Ig-like domains and two cytoplasmic immunoreceptor tyrosine-based inhibitory motifs, are considered to be functional orthologs of Human Siglec-9^36^. Siglec-E-deficient mouse macrophages exhibit enhanced pro-inflammatory cytokine secretion and bactericidal activity against infection by group B Streptococcus^37^. The extracellular domain of human sSiglec-9 versus mouse and rat sSiglec-E are 56.88% and 57.36% identical, respectively. While rodent Siglec-E is important in the immune response, no role has been reported for its secreted ectodomain, which we identified in SHED-CM. We previously showed that MCP-1 and sSiglec-9 induce M2 polarization through the MCP-1 receptor CCR2. sSiglec-9 binds to sialylated carbohydrates on CCR2 and may modify the MCP-1/CCR2-induced signaling^14^. In the present study, we showed that treating macrophages with the selective CCR2 antagonist RS504393 inhibited the MCP-1/sSiglec-9-induced expression of M2 markers and multiple trophic factors for liver regeneration.

The present study has a few limitations that should be addressed in the future. First, we found that MCP-1/sSiglec-9 suppressed the apoptosis of hepatocytes *in vivo* and *vitro*. To reveal the molecular mechanisms underlying MCP-1/sSiglec-9’s therapeutic effect, we need to determine how this treatment suppresses hepatocyte apoptosis, including the possible roles of MAP kinase cascades. Second, we used a D-gal-induced acute hepatitis model to investigate the effect of MCP-1/sSiglec-9 in the present study. D-Gal induces lethal hepatitis accompanied by a hepatic inflammatory response and the generation of endogenous LPS from gut microbiota in the animal’s own intestines^38^,^39^. However, there are some differences in the mechanism of liver injury between the D-Gal-induced and other models, such as ConA-, acetaminophen-, or carbon tetrachloride-induced hepatitis. It will be important to assess whether the M2 induction by MCP-1/sSiglec-9 shows a therapeutic effect in all of the above models.

In conclusion, we found that MCP-1 and sSiglec-9 synergistically induced M2 macrophage polarization via CCR2, and that this population of M2 macrophages produced hepatoregenerative factors. Furthermore, we found that a single intravenous administration of MCP-1/sSiglec-9 suppressed the inflammatory response, inhibited hepatocyte apoptosis, promoted hepatocyte proliferation, and improved the survival rate in an ALF rat model. Our data suggest that the unique combination of MCP-1 and sSiglec-9 may provide therapeutic benefits for patients with ALF.

## Methods

### Animals

Seven- to nine-week-old female Sprague-Dawley rats weighing 200–230 g from Japan SLC (Shizuoka, Japan) were used for the ALF experiments. The animal studies were carried out in accordance with the NIH Guidelines for the Care and Use of Laboratory Animals. The experimental protocol was approved by the Institutional Animal Care and Use Committee of Nagoya University (26187, 28282).

### Isolation of stem cells from human deciduous teeth (SHEDs)

SHEDs were isolated as previously described^17^. In brief, exfoliated deciduous teeth (from 6- to 12-year-old individuals), extracted for clinical purposes, were collected at Nagoya University Graduate School of Medicine using approved guidelines set by Nagoya University (H-73, 2003). All methods were performed in accordance with this guideline. Ethical approval was obtained from the ethics committee of Nagoya University (permission number 8–2). All participants provided written informed consent. After the crown and root were separated, the dental pulp was collected and then digested in a solution containing 3 mg/ml collagenase type 1 and 4 mg/ml dispase for 1 h at 37 °C. Single-cell suspensions (1–2 × 10^4^ cells/ml) were plated on culture dishes in DMEM supplemented with 10% fetal calf serum, and then incubated at 37 °C in 5% CO_2_.

### Preparation of SHED-CM and depletion of MCP-1 and sSiglec-9.

At passage 3–9, SHEDs at 70–80% confluency were washed with PBS, and the culture medium was replaced with serum-free DMEM. After a 48-h incubation, the medium was collected and centrifuged for 3 min at 440 × g. The supernatants were collected and centrifuged for 3 min at 4 °C and 17,400 × g. The second supernatant was collected and used as the CM in subsequent experiments. To deplete MCP-1 and sSiglec-9 from the SHED-CM, Protein-G Sepharose (GE Healthcare, Piscataway, NJ), pre-bound with anti-MCP-1 and anti-Siglec-9 antibodies was added to the SHED-CM. The mixture was incubated for 1 h at 4 °C, and the antibody beads were removed by centrifugation. The depletion procedure was repeated 3 more times, and the MCP-1 and sSiglec-9 depletion was confirmed by ELISA (Human CCL2/MCP-1 ELISA Kit, R&D Systems, Minneapolis, MN; Human Siglec-9 ELISA Kit, Ray Biotech, Norcross, GA). The depleted CM was called dSHED-CM.

### Acute liver failure induction and treatment

After the rats were anesthetized, a fresh solution of D-Gal, dissolved in physiological saline and adjusted to pH 7.3 with 1 N NaOH, was intraperitoneally injected at 1.2 g/kg. Our preliminary studies indicated that this model was associated with high mortality rates. Twenty-four hours after D-Gal injection, (1) SHED-CM, dSHED-CM, or an equivalent volume of serum-free DMEM as a control, or (2) recombinant human MCP-1 (MCP-1; 279-MC; R&D Systems) only (1 μg/ml), recombinant human secreted ectodomain of Siglec-9 (sSiglec-9; 1139-SL; R&D Systems) only (1 μg/ml), or an MCP-1/sSiglec-9 mixture (1 μg/ml, each) in 1 ml PBS, or an equivalent volume of PBS, was injected into the tail vein. Ten animals per group were used to assess the survival rates, and 5 animals per group were collected at 0 (sham), 24 (pre-treatment, Pre), 36, 48, 60, 72, and 96 h after D-Gal injection for liver enzyme analyses. In addition, four animals per group were sacrificed for tissue collection at 0, 24 (Pre), and 36 h (PBS- and MCP-1/sSiglec-9-treated groups) after D-Gal infusion (Fig. 1a).

### Serum cytokine levels

Interleukin (IL)-1α and IL-10 serum levels at 0 (sham), 24 (Pre), and 48 h after D-Gal injection were measured using the Cytometric Bead Array (BD^TM^CBA, BD Biosciences, Franklin Lakes, NJ).

### Real-time quantitative (q)-PCR

Total RNA was quantified by a spectrophotometer, and RNA integrity was checked on 1% agarose gels. Reverse transcription reactions were performed with Superscript III Reverse Transcriptase (Invitrogen, Carlsbad, CA) using 0.5 μg total RNA in a 25-μl total reaction volume. Real-time q-PCR was performed using the THUNDERBIRD SYBR qPCR Mix (Toyobo, Osaka, Japan) and the StepOnePlus Real-Time PCR System (Applied Biosystems, Foster City, CA). Rat primers were designed using primer 3 (Supplementary Table S5). The obtained results for each animal were normalized to GAPDH, and then the normalized value was compared to that obtained in the sham experiment to determine the relative mRNA expression.

### Liver histopathology

For histological examination, the animals were anesthetized and sacrificed 0 (sham), 24 (Pre), and 36 h after D-Gal injection. Formalin-fixed, paraffin-embedded liver samples were cut into 4-μm-thick sections and stained with hematoxylin-eosin (HE).

### Immunohistochemical analysis

For immunohistochemical examination of the livers, the animals were anesthetized and sacrificed 36 h after D-Gal infusion. The livers were removed and embedded in OCT compound (Sakura Finetek Japan, Tokyo, Japan) and cut into 4-μm-thick sections on a cryostat. The sections were then permeabilized with 0.1% (v/v) Triton X-100 in PBS for 20 min, blocked with 5% (v/v) bovine serum albumin for 30 min, and incubated overnight with the following primary antibodies purchased from Abcam (Cambridge, U.K.): anti-Arginase-1 (goat IgG, 1:50), anti-iNOS (rabbit IgG, 1:50), anti-CD11b (mouse IgG, 1:300), anti-Albumin (chicken IgG, 1:400), and anti-Ki-67 (mouse IgG, 1:300). The following secondary antibodies were purchased from Invitrogen: anti-mouse IgG–Alexa Fluor 488, anti-rabbit IgG–Alexa Fluor 546, anti-goat IgG–Alexa Fluor 546, anti-rabbit IgG–Alexa Fluor 647, and streptavidin-conjugated Alexa Fluor 647. Anti-chicken IgY-Alexa Fluor 488 was from Abcam. After counterstaining with DAPI (Sigma-Aldrich, St. Louis, MO), tissue images were captured with a confocal laser scanning microscope (TiE-A1R-KT5, Nikon, Tokyo, Japan). Apoptotic cell death was analyzed by terminal deoxynucleotidyl transferase dUTP nick end labeling (TUNEL) assay (In Situ Cell Death Detection Kit, Roche, Basel, Switzerland). The average number of TUNEL-, Ki-67-, Arginase1-, iNOS- and CD11b-positive cells was determined by counting 45 random fields under a universal fluorescence microscope (BZ9000, Keyence, Osaka, Japan); at least 3 animals per group were examined.

### Macrophage isolation and activation

Bone marrow cells were isolated from the femurs and tibias of 6- to 8-week-old female Sprague-Dawley rats. They were plated in 60-mm cell culture dishes (2.0 × 10^6^ cells per dish) and differentiated into bone marrow macrophage (BMM) lineages in DMEM supplemented with 20 ng/ml macrophage colony-stimulating factor (Peprotech, NJ, USA) at 37 °C in 5% CO_2_ for 7–8 d. Next, they were incubated in serum-free DMEM with 100 ng/ml MCP-1 and 100 ng/ml sSiglec-9, (MCP-1/sSiglec-9), 20 ng/ml IL-4 (R&D Systems), or serum-free DMEM. After a 48 h incubation, the cells’ morphologies and mRNA expression patterns were examined, and the conditioned media were collected and used as macrophage CMs [M(MCP-1s/Siglec-9)-CM, M(IL-4)-CM, and M(DMEM)-CM] in the hepatocyte protection assays below. To determine the effect of CC chemokine receptor 2 (CCR2) inhibition on M2 differentiation, the BMMs were incubated for 30 min at 37 °C in 5% CO_2_ with serum-free DMEM containing 50 mM RS504393 (6-methyl-1′-[2-(5-methyl-2-phenyl-4-oxazolyl)ethyl]-spiro [4 *H*-3,1-benzoxazine-4,4′-piperidin]-2(1*H*)-one; Tocris Bioscience, Avonmouth, UK) before adding MCP-1/sSiglec-9.

### Hepatocyte protection assay

To obtain hepatocytes, a rat liver was perfused with liver perfusion medium (Life Technologies, Palo Alto, CA) at a flow rate of 3 ml/min for 5 min, followed by perfusion with a basic perfusion solution (136 mM NaCl, 5.4 mM KCl, 5 mM CaCl2, 0.5 mM NaH_2_PO_3_, 0.42 mM Na_2_HPO_3_·12H_2_O, 10 mM HEPES pH 7.5, 5 mM glucose, and 4.2 mM NaHCO_3_) containing 0.5 g/l collagenase type 4 (Yakult, Tokyo, Japan) at a flow rate of 3 ml/min for 8 min. The digested liver was transferred to glass dishes containing DPBS (Dulbecco’s phosphate-buffered saline) and chopped into small pieces using a surgical knife. The extracted cells were dispersed by pipetting and were passed through a 70-μm cell strainer. After centrifugation at 50 × g for 4 min, the cell pellet was suspended in an iso-osmotic Percoll solution and centrifuged at 70 × g for 10 min. The pellet was then washed with DMEM, and single-cell suspensions (1–2 × 10^6^ cells/ml) were plated on a Collagen-1-coated 48-well tissue-culture plate. Hepatocyte apoptosis was induced using 2.5 mM D-Gal and 5 μg/ml LPS in the presence of serum-free DMEM only, DMEM with MCP-1 and sSiglec-9 (100 ng/ml, each), M(DMEM)-CM, M(IL-4)-CM, or M(MCP-1/sSiglec-9)-CM for 24 h. Each experiment consisted of triplicate assays, and was confirmed in three separate experiments.

### M2 macrophage depletion assay *in vivo*

To deplete M2 macrophages, 1 ml of clodronate encapsulated inside mannosylated liposomes (m-Clodrosome: 5 mg/ml Clodronate Disodium Salt, 18.8 mg/ml L-α-Phosphatidylcholine, 4.2 mg/ml Cholesterol, and 1 mg/ml 4-Aminophenyl-alpha-D-Mannopyranoside; Encapsula Nano Sciences, Brentwood, TN, United States) was intravenously administered together with MCP-1/sSiglec-9. Control rats received plain mannosylated liposomes (m-Encapsome: 18.8 mg/ml L-α-Phosphatidylcholine, 4.2 mg/ml Cholesterol, and 1 mg/ml 4-Aminophenyl-alpha-D-Mannopyranoside) in the same volume. m-Clodrosome depletes M2 macrophages, which express a mannose receptor (MR, MMR, or CD206) or SIGNR1. Five animals per group were sacrificed for tissue collection 36 h after D-Gal infusion.

### Statistical analysis

The SPSS software package, version 22.0 (IBM, New York, NY, USA) was used for statistical analyses. An actuarial analysis of the cumulative survival rates was performed using the Kaplan–Meier method, and differences across groups were compared using the log-rank test. All data are expressed as the mean ± standard error of the mean (SEM). Differences between groups were compared using the Student’s t test or Mann-Whitney U-test, according to the data type. Differences in serum cytokines before and after treatments were compared using the Wilcoxon signed-rank test. Statistical significance was defined as *p* < 0.05.

## Additional Information

**How to cite this article:** Ito, T. *et al*. Secreted Ectodomain of SIGLEC-9 and MCP-1 Synergistically Improve Acute Liver Failure in Rats by Altering Macrophage Polarity. *Sci. Rep.* **7**, 44043; doi: 10.1038/srep44043 (2017).

**Publisher's note:** Springer Nature remains neutral with regard to jurisdictional claims in published maps and institutional affiliations.

## Supplementary Material

Supplementary Information
