# Supplementary material for: Secreted Ectodomain of SIGLEC-9 and MCP-1 Synergistically Improve Acute Liver Failure in Rats by Altering Macrophage Polarity
Source: Sci Rep. 2017 Mar 8;7:44043. doi: 10.1038/srep44043 (PMC5358744; doi:10.1038/srep44043)
Supplement: Supplementary Information [file srep44043-s1.pdf]

Supplementary Information

Secreted Ectodomain of SIGLEC-9 and MCP-1 Synergistically Improve Acute Liver Failure in Rats by Altering Macrophage Polarity

Takanori Ito[1], Masatoshi Ishigami[1], Yoshihiro Matsushita[2], Marina Hirata[2], Kohki Matsubara[2], Tetsuya Ishikawa[1], Hideharu Hibi[2], Minoru Ueda[2], Yoshiki Hirooka[1] Hidemi Goto[1] and Akihito Yamamoto*[2,3]

[1] Department of Gastroenterology and Hepatology,

[2] Department of Oral and Maxillofacial Surgery of Nagoya University Graduate School of Medicine, 65 Tsurumai-cho, Showa-ku, Nagoya 466-8550, Japan.

[3] Department of Oral histology, Institute of Biomedical Science, Tokushima University Graduate School, 3-18-5 Kuramoto-cho, Tokushima 770-8504, Japan

Contact information/Correspondence

Akihito Yamamoto

akihito@tokushima-u.ac.jp

3-18-5 Kuramoto-cho, Tokushima

Supplementary Table S1.

MCP-1 and sSiglec-9 levels in SHED-CM and in MCP-1- and sSiglec-9-depleted SHED-CM

	**MCP-1 (pg/ml)**	**sSiglec-9 (pg/ml)**
**SHED-CM**	317.3±3.8*	569.1±3.3
**d-SHED-CM**	18.3±1.0	ND

MCP-1 and sSiglec-9 levels in SHED-CM and d-SHED-CM were measured by ELISA. Data represent the mean ± SEM. ND, not detected. * $p < 0.05$. (SHED-CM vs dSHED-CM)

Supplementary Table S2.

The numbers of TUNEL or Ki-67 / DAPI positive-stained hepatocytes in Figure3

	TUNEL	DAPI
**DMEM**	539 (374 - 905)	749 (609 - 1199)
**MCP-1/sSiglec-9**	430 (308 - 577)	638 (589 - 709)
**M(DMEM)-CM**	540 (400 - 721)	673 (557 - 868)
**M(IL-4)-CM**	471 (288 - 675)	660 (534 - 865)
**M(MCP-1/sSiglec-9)-CM**	138 (23 - 288)	630 (376 - 985)
	**Ki-67**	**DAPI**
**DMEM**	94 (0 - 184)	571 (268 - 756)
**MCP-1/sSiglec-9**	98 (48 - 152)	619 (528 - 796)
**M(DMEM)-CM**	142 (20 - 344)	587 (372 - 864)
**M(IL-4)-CM**	107 (20 - 216)	421 (213 - 700)
**M(MCP-1/sSiglec-9)-CM**	456 (260 - 920)	661 (396 - 1272)

Values are expressed as means (range).

Supplementary Table S3.

The numbers of TUNEL / DAPI and Ki-67 positive-stained cells in Figure4

	TUNEL	DAPI
**PBS**	472 (242 - 667)	802 (668 - 927)
**MCP-1/sSiglec-9**	37(19 - 59)	822 (536 - 1010)
	**Ki-67**	
**PBS**	110 (35 - 224)	
**MCP-1/sSiglec-9**	281 (231 - 338)	

Values are expressed as means (range).

Supplementary Table S4.

The numbers of iNOS or Arginase-1 / CD11b positive-stained cells in Figure6

	iNOS	CD11b
**PBS**	15 (9 - 37)	22 (11 - 38)
**MCP-1/sSiglec-9**	3 (0 - 8)	31 (20 - 50)
	**Arginase-1**	**CD11b**
**PBS**	3 (0 - 7)	16 (4 - 30)
**MCP-1/sSiglec-9**	20(10 - 35)	25 (12 - 41)

Values are expressed as means (range).

Supplementary Table S5.

Rat primers for real time q-PCR

Primer	Sequence (forward 5'-3')	Sequence (reverse 5'-3')
Gapdh	AACTTTGGCATCGTGGAAGG	CGGATACATTGGGGGTAGGA
Il-6	TTGCCTTCTTGGGACTGATG	ACTGGTCTGTTGTGGGTGGT
Il-1β	CAGGATGAGGACCCAAGCAC	TCAGACAGCACGAGGCATTT
Tnf-α	CTCGAGTGACAAGCCCGTAG	CCTTGAAGAGAACCTGGGAGTAG
iNos	GGCAGGATGAGAAGCTGAGG	CCGCATTAGCACAGAAGCAA
Caspase-1	GGAGCTTCAGTCAGGTCCATC	CGCCACCTTCTTTGTTCAGTTT
Il-10	GCCTGCTCTTACTGGCTGGA	TCTGGCTGACTGGGAAGTGG
Tgf-β	CCGCAACAACGCAATCTATG	GCACTGCTTCCCGAATGTCT
Ym-1	TGCCAACATCAGCAACAACA	CCATCCTCCAACAGACAGCA
Cd206	GCAGGTGGTTTATGGGATGTTT	TTTGGGTTCAGGAGTTGTTGTG
Arg-1	CACCTGAGTTTTGATGTTGATGG	TCCTGAAAGTAGCCCTGTCTTGT
Ccr2	AGAGGCATAGGGCTGTGAGG	CCTGGAAGGTGGTCAGGAAG
Cd11b	TACCGGAAGGTGTCAGCAAG	TTAGCGGGAAAGATGGGATG
Hgf	GCAAGACATGTCAGCGCTGG	CCAAGGGGTGTCAGGGTCAA
Vegf	ACCAAAGCCAGCACATAGGA	GGGGCATTAACTGCATCTGG
Igf	GCTGTGTAAACGACCCGGGA	ACTGAAGAGCGTCCACCAGC

Supplementary Figure S1.

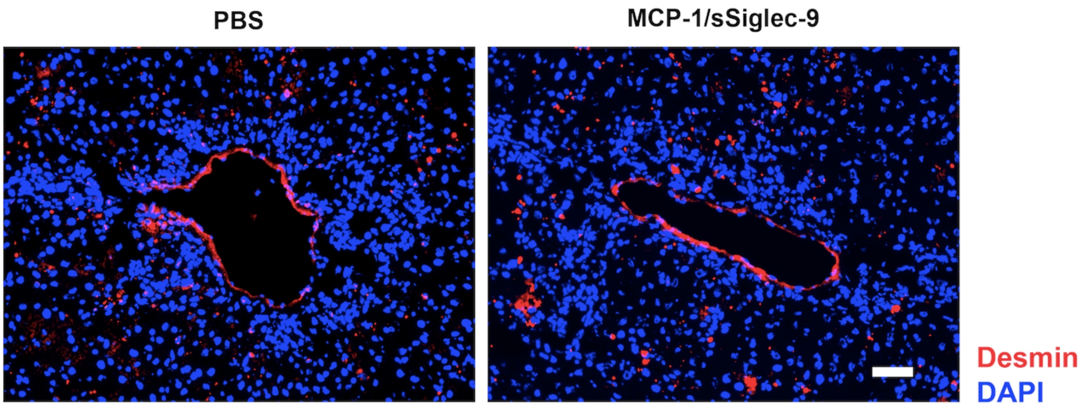

MCP-1 and sSiglec-9 have little or no effect on the activation of hepatic stellate cells

Representative images of Desmin immunofluorescence staining of the livers, 12 h after MCP-1/sSiglec-9 or PBS injection. Scale bar: 50 μm.
